# Machine-learning-based Web system for the prediction of chronic kidney disease progression and mortality

**DOI:** 10.1371/journal.pdig.0000188

**Published:** 2023-01-18

**Authors:** Eiichiro Kanda, Bogdan Iuliu Epureanu, Taiji Adachi, Naoki Kashihara

**Affiliations:** 1 Medical Science, Kawasaki Medical School, Kurashikishi, Okayamaken, Japan; 2 College of Engineering, University of Michigan, Ann Arbor, Michigan, United States of America; 3 Department of Biosystems Science, Institute for Life and Medical Sciences, Kyoto University, Kyotoshi, Kyotofu, Japan; 4 Department of Nephrology and Hypertension, Kawasaki Medical School, Kurashikishi, Okayamaken, Japan; Dalhousie University, CANADA

## Abstract

Chronic kidney disease (CKD) patients have high risks of end-stage kidney disease (ESKD) and pre-ESKD death. Therefore, accurately predicting these outcomes is useful among CKD patients, especially in those who are at high risk. Thus, we evaluated whether a machine-learning system can predict accurately these risks in CKD patients and attempted its application by developing a Web-based risk-prediction system. We developed 16 risk-prediction machine-learning models using Random Forest (RF), Gradient Boosting Decision Tree, and eXtreme Gradient Boosting with 22 variables or selected variables for the prediction of the primary outcome (ESKD or death) on the basis of repeatedly measured data of CKD patients (n = 3,714; repeatedly measured data, n = 66,981) in their electronic-medical records. The performances of the models were evaluated using data from a cohort study of CKD patients carried out over 3 years (n = 26,906). One RF model with 22 variables and another RF model with 8 variables of time-series data showed high accuracies of the prediction of the outcomes and were selected for use in a risk-prediction system. In the validation, the 22- and 8-variable RF models showed high C-statistics for the prediction of the outcomes: 0.932 (95% CI 0.916, 0.948) and 0.93 (0.915, 0.945), respectively. Cox proportional hazards models using splines showed a highly significant relationship between the high probability and high risk of an outcome (*p*<0.0001). Moreover, the risks of patients with high probabilities were higher than those with low probabilities: 22-variable model, hazard ratio of 104.9 (95% CI 70.81, 155.3); 8-variable model, 90.9 (95% CI 62.29, 132.7). Then, a Web-based risk-prediction system was actually developed for the implementation of the models in clinical practice. This study showed that a machine-learning-based Web system is a useful tool for the risk prediction and treatment of CKD patients.

## Author summary

Chronic kidney disease (CKD) patients have high risks of end-stage kidney disease (ESKD) and pre-ESKD death. Although the development of a new artificial intelligence (AI) model is expected to be useful for screening CKD patients at high risk, none of the models developed so far have yet been put into practical use. There are many reasons for the difficulties in developing and applying AI models to clinical settings: (1) AI models often have too many variables, some of which are uncommon, to input in busy clinical settings. (2) The accuracy of AI models to predict risks is not always high when evaluating nontarget patients’ prognoses. (3) The codes of AI models do not always match electronic medical records of different hospitals. Therefore, in this study, AI models with a small number of commonly used variables were developed. Then, their performances were rigorously evaluated on subgroups of CKD patients classified on the basis of kidney function, age, and diabetes mellitus. Moreover, we developed and opened a Web-based risk-prediction system using the selected AI models that showed the highest accuracies for clinical practice: http://160.16.88.112:8000/. Our study fills the gap between AI development in research and AI in clinical practice.

## Introduction

Chronic kidney disease (CKD) patients have high risks of end-stage kidney disease (ESKD), cardiovascular disease (CVD), and death [1–3]. With the progression of CKD, the risks of these outcomes increase. In addition, comorbidities of CKD, such as CKD-mineral and bone disorder (MBD), renal anemia, and hyperpotassemia, also appear with CKD progression [4–9]. Moreover, the medical costs of CKD therapy are high: CKD imposes heavy physical and economic burdens on patients [10,11]. To slow CKD progression, control of the various risk factors, such as diabetes, hypertension, and proteinuria, is needed [12].

Kidney Disease: Improving Global Outcomes (KDIGO) 2012 Clinical Practice Guideline for the Evaluation and Management of CKD proposed a classification system to diagnose CKD, recommended referral to specialists, and provided models of CKD care [4]. Accurate prognoses of a patient’s renal function and life expectancy are useful for diagnosing CKD patients with a high risk of ESKD and determining therapeutic strategies for them [4,5]. Thus, an easily accessible risk-prediction system would be useful for these purposes.

The risk prediction of an outcome, such as ESKD, is evaluated using the probabilities of the outcome occurring, as predicted using clinical indices on the basis of statistical models [13–15]. Probabilities are useful as risk indices in clinical settings for risk estimation, diagnosis, clinical therapy decision, and specialist referral, among others [16]. Machine-learning prediction is also based on probability. Various machine-learning models for the evaluation of CKD progression have been developed [17–21]. Although the development of a new risk-prediction system using machine learning is expected to be useful for CKD therapy, none of these models have yet been put into practical use.

There are many reasons for the difficulties in developing and applying machine-learning models to clinical settings. For example, (1) machine-learning models often have too many variables to input and uncommon variables at clinical settings. (2) The accuracy of machine-learning models to predict risks is not always high when evaluating nontarget patients’ prognoses. (3) The code developed for machine learning does not always match electronic medical records (EMRs) of different hospitals. Moreover, developing a risk-prediction system requires the knowledge of not only medicine but also different fields of data-science engineering, such as machine learning, servers, and networks.

Therefore, this study was conducted to clarify whether the probability of the occurrence of either ESKD or pre-ESKD death in CKD patients can be accurately predicted using machine-learning models with a small number of commonly used variables. Then, the applicability of the models to a subclass of patients was evaluated on the basis of estimated glomerular filtration rate (eGFR), age, and diabetes mellitus (DM) using data from a cohort study of CKD patients. Moreover, we developed a Web-based risk-prediction system for the implementation of the models in clinical practice.

## Results

### Model selection

#### Comparison of C-statistics among models

**Fig 1:**
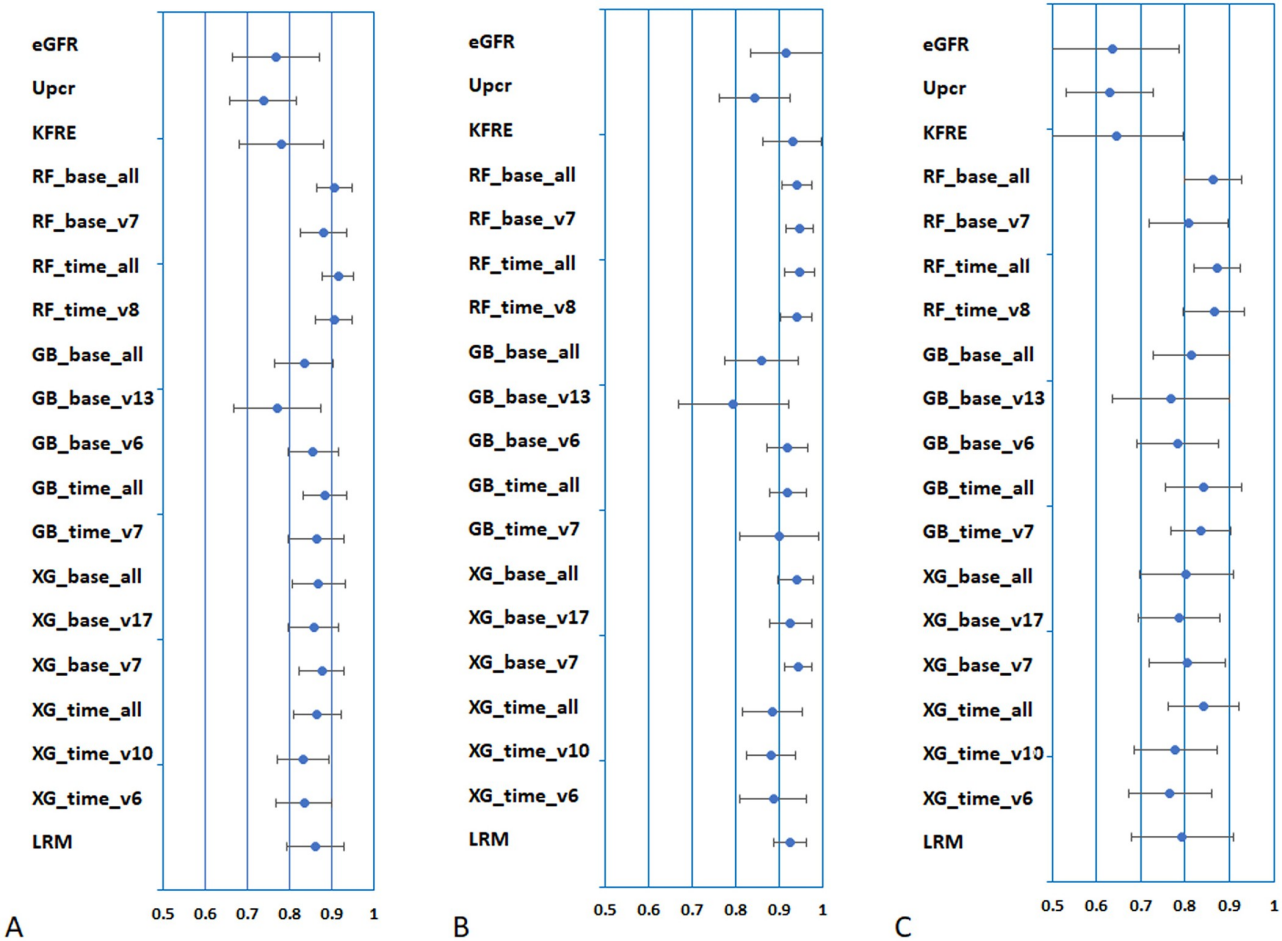
Model performances for prediction in outcomes over 1 year at model selection stage. Figures show the C-statistics for the prediction of outcomes with 95% CIs of the models. A: Primary outcome. B: ESKD. C: Death. Abbreviations: ESKD, end-stage kidney disease; eGFR, estimated glomerular filtration rate; UPCR, urinary protein-to-creatinine ratio; KFRE, kidney failure risk equation; RF, Random Forest; GB, Gradient Boosting Decision Tree; XG, eXtreme Gradient Boosting; LRM, logistic regression model; CI, confidence interval.

In this study, the models were denoted by their algorithm, dataset type (baseline or time-series dataset), and number of variables (all or v+number). For example, a Random Forest (RF) model based on the baseline dataset including x variables was denoted RF_base_vx. Fig 1A shows the C-statistics of the models used for predicting the primary outcome (ESKD or death) over 1 year. The C-statistics of RF_time_all and RF_time_v8 were higher than 0.85 (*p*<0.05). The C-statistics for the prediction of ESKD over 1 year showed high accuracy for most of the models (Fig 1B). In particular, the C-statistics of 5 models were more than 0.9 for the prediction over 1 year (*p*<0.05). Moreover, these models exhibited high C-statistics for the prediction of death over 1 year (Fig 1C). Similarly, the models showed high prediction accuracies for the primary outcome over 2 and 3 years (S1 Fig).

#### Comparison of model performance in subclasses

The C-statistics of the primary outcome were also compared among models for subclasses (S2 Fig). In each subclass, the models showed high C-statistics. S1 Table summarizes the applicability of the models to patients with various conditions. Most of the models with all the variables provided high C-statistics. In particular, RF_base_all, RF_time_all, and RF_time_v8 showed high performances. Considering these results and the calculation speed of the server, RF_time_all and RF_time_v8 were selected as representative models for the development of a Web-based risk-prediction system.

**Fig 2:**
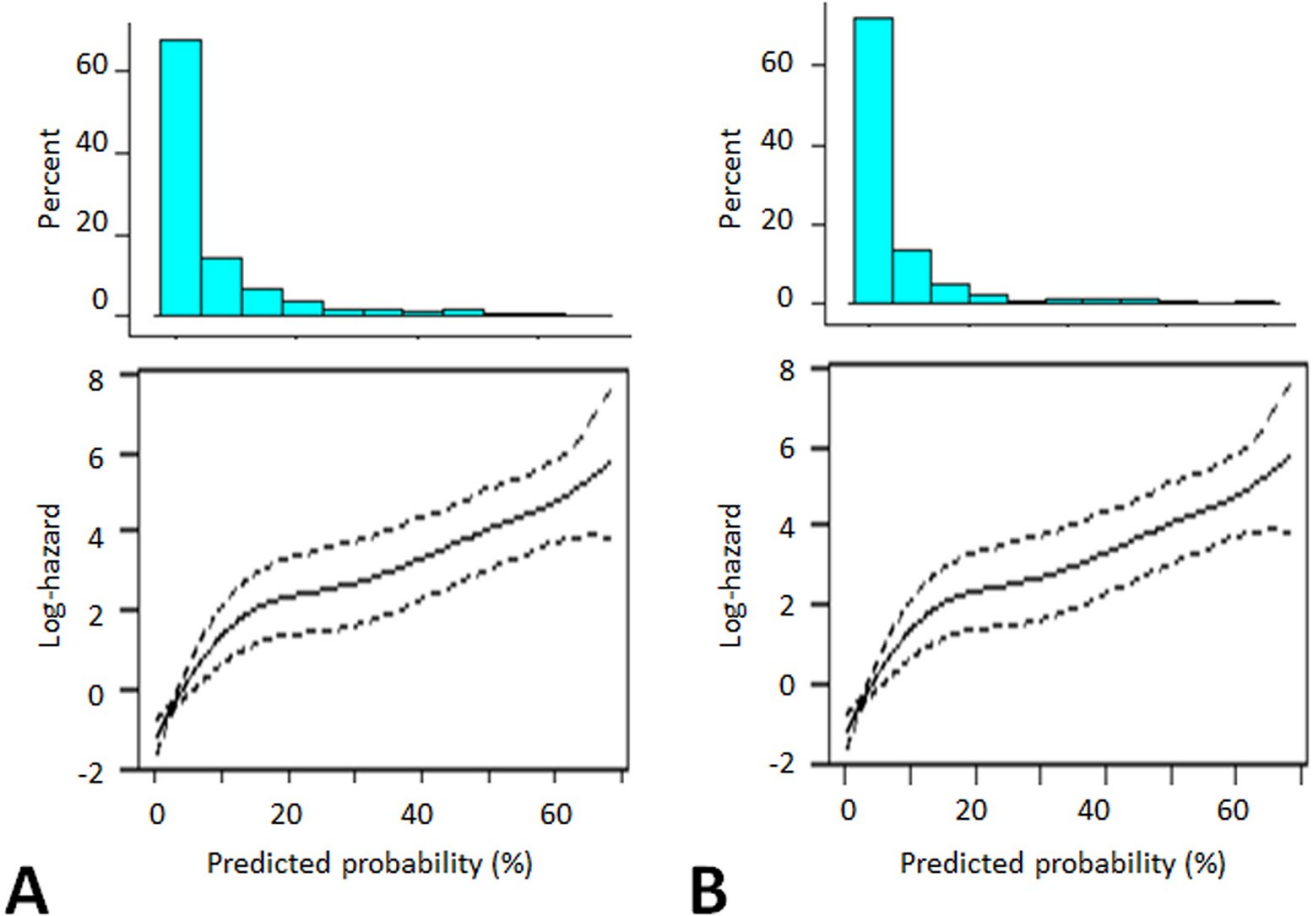
Relationships between risk of primary outcome and its predicted probabilities at model selection stage. The figure shows the relationships between the risk of the primary outcome and its predicted probabilities. Histograms of the probabilities and the log hazards of the primary outcome are shown in the upper and lower panels, respectively. A: RF_time_all. B: RF_time_v8. Abbreviation: RF, Random Forest.

The relationship between the risk of the primary outcome and its predicted probabilities was analyzed (Fig 2). The hazard ratios (HRs) of RF_time_all and RF_time_v8 were associated with high probabilities (*p*<0.0001). For the models to be informative and show whether a patient was at a high risk, the cutoff levels of the predicted probability were 8.9% for RF_time_all and 8.2% for RF_time_v8.

### Validation of the machine-learning models using CKD cohort study data

Baseline characteristics and outcome events of the validation dataset are shown in S2 Table and S3 Table. The mean±SD and median (IQR) of the probability predicted by the models were 2.5±5.6, 1.0 (0.6, 1.9)% for RF_time_all and 2.2±6.0, 0.6 (0.3, 1.3)% for RF_time_v8.

S4 Table summarizes the applicability of the models to patients with various conditions. RF_time_all and RF_time_v8 showed high C-statistics in the cohort study data. Their high C-statistics for the prediction of the primary outcome over 3 years were 0.932 95% confidence intervals (CIs) (0.916, 0.948) and 0.93 (0.915, 0.945). These values were statistically significantly higher than that using obtained the kidney-failure-risk equation (KFRE) 0.905 (0.881, 0.929; *p* = 0.035, 0.033, respectively).

For the prediction of ESKD over 3 years, the models showed high C-statistics of more than 0.95: 0.966 (95% CI 0.956, 0.976), 0.966 (95% CI 0.957, 0.975). For the prediction of death over 3 years, the C-statistics of the models were higher than 0.8: 0.859 (95% CI 0.821, 0.898), 0.854 (95% CI 0.817, 0.89).

**Fig 3:**
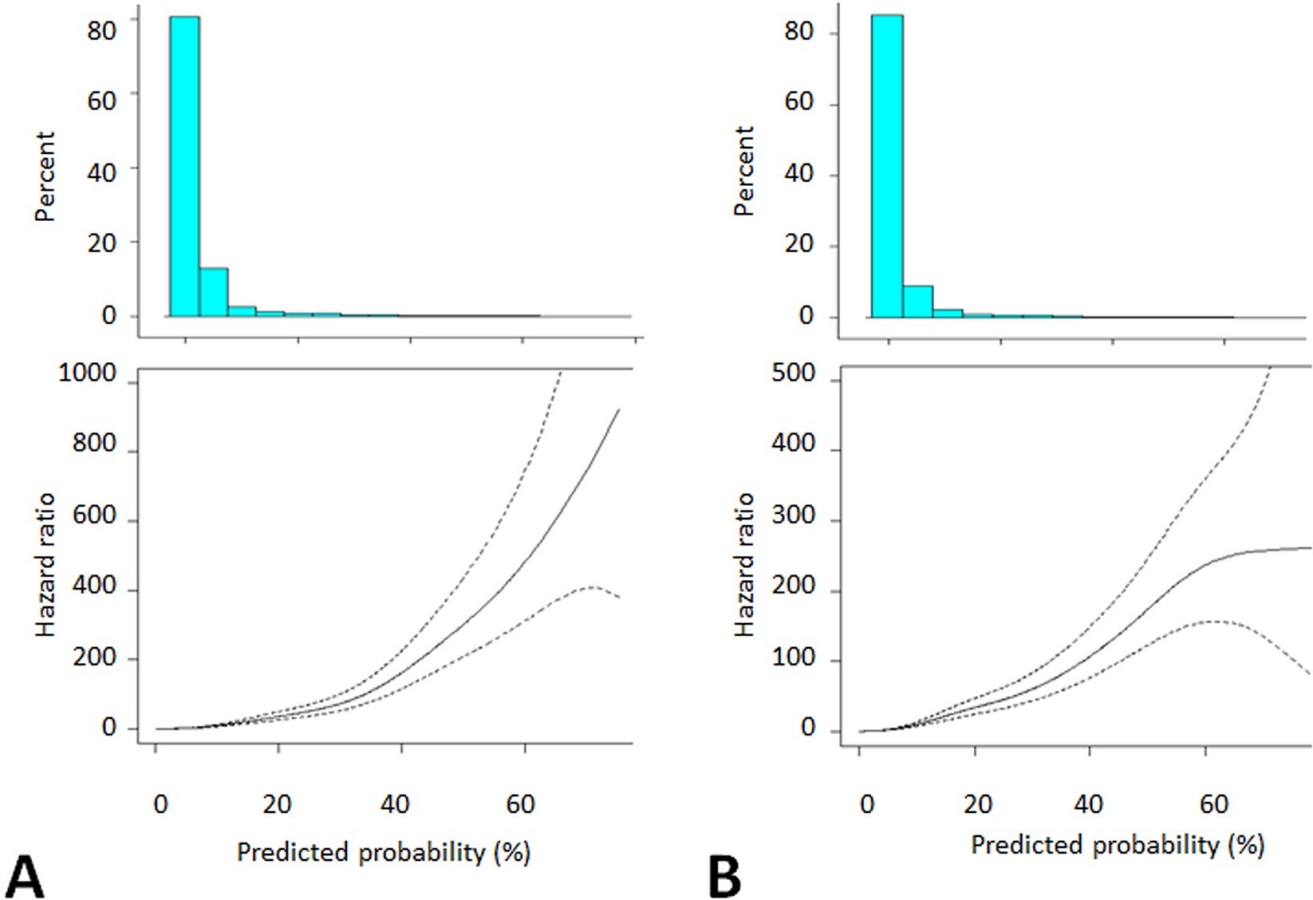
Relationships between risk of primary outcome and its predicted probability. Histograms of the probabilities and the hazard ratios of the primary outcome are shown in the upper and lower panels, respectively. A, RF_time_all, *p*<0.0001; B, RF_time_v8, *p*<0.0001. Abbreviations: HR, hazard ratio; RF, Random Forest.

Cox proportional hazards models showed the relationships between high probabilities predicted using the models and high risks of the primary outcome on the basis of the follow-up period (Fig 3).

#### Risk of outcome events and high-risk patients

Among the patients, 5.5% were classified into the high-risk groups on the basis of the cutoff levels of RF_time_all and RF_time_v8, and they showed different characteristics from the low-risk group (Table 1).

**Table 1:** Baseline characteristics of risk groups classified on the basis of the results of machine-learning models.

Model names	RF_time_all			RF_time_v8		
Risk groups	Low-risk group	High-risk group	*p* value	Low-risk group	High-risk group	*p* value
N (%)	25,422 (94.5)	1,484 (5.5)		25,428 (94.5)	1,478 (5.5)	
**Demographic characteristics**						
Age (years)	61.2±16.3	62.0±17.9	0.056	61.2±16.3	62.2±18.1	0.022
Male (%)	13,000 (51.1)	779 (52.5)	0.31	13,020 (51.2)	759 (51.4)	0.92
Comorbidities						
DM (%)	5,305 (20.9)	332 (22.4)	0.17	5,293 (20.8)	344 (23.3)	0.025
Hypertension (%)	5,234 (20.6)	336 (22.6)	0.060	5,230 (20.6)	340 (23.0)	0.027
CVD (%)	159 (0.6)	30 (2.0)	<0.0001	162 (0.6)	27 (1.8)	<0.0001
**Laboratory data**						
eGFR (mL/min/1.73m^2^)	73.3±31.0	68.7±26.4	<0.0001	73.3±31.0	69.2±29.7	<0.0001
Albumin (g/dL)	4.2±0.5	4.0±0.63	<0.0001	4.2±0.5	4.0±0.6	<0.0001
Sodium (mmol/L)	140.3±2.6	139.9±3.0	<0.0001	140.3±2.6	139.9±300	<0.0001
Potassium (mmol/L)	4.2±0.4	4.2±0.5	0.74	4.2±0.4	4.2±0.5	0.16
Calcium (mg/dL)	9.2±0.5	9.0±0.7	<0.0001	9.2±0.5	9.1±0.7	<0.0001
Phosphorus (mg/dL)	3.4±0.7	3.5±1.0	0.028	3.4±0.7	3.5±0.9	0.008
LDL (mg/dL)	111.0±32.1	110.6±36.5	0.71	111.0±32.1	110.0±36.7	0.32
Uric acid (mg/dL)	5.2±1.5	5.3±1.6	0.54	5.2±1.5	5.3±1.6	0.40
WBC (10^3^/μL)	6.3±3.0	6.6±4.1	<0.0001	6.3±3.0	6.6±4.1	<0.0001
Hemoglobin (g/dL)	13.6±1.8	13.1±2.3	<0.0001	13.6±1.8	13.1±2.3	<0.0001
UPCR (g/gCre)	0.27 [0.11, 1.00]	0.43 [0.14, 2.47]	<0.0001	0.27 [0.11, 1.00]	0.42 [0.14, 2.42]	<0.0001
**Medications**						
RAASI (%)	4,407 (17.3)	268 (18.1)	0.48	4,404 (17.3)	271 (18.3)	0.32
Phosphorus absorbent (%)	209 (0.8)	52 (3.5)	<0.0001	214 (0.8)	47 (3.2)	<0.0001
Vitamin D (%)	653 (2.6)	63 (4.2)	<0.0001	653 (2.6)	63 (4.3)	<0.0001
Statin (%)	4,162 (16.4)	180 (12.1)	<0.0001	4,145 (16.3)	197 (13.3)	0.002
Uric-acid-lowering medicines (%)	2,272 (8.9)	174 (11.7)	<0.0001	2,286 (9.0)	160 (10.8)	0.020
ESA (%)	665 (2.6)	82 (5.5)	<0.0001	675 (2.7)	72 (4.9)	<0.0001

Continuous variables are shown as mean±SD or median (interquartile range). Categorical variables are shown as n (%).Abbreviations: DM, diabetes mellitus; CVD, cardiovascular disease; eGFR, estimated glomerular filtration rate; LDL, low-density lipoprotein; WBC, white blood cells; UPCR, urinary protein-to-creatinine ratio; RAASI, renin-angiotensin-aldosterone system inhibitor; ESA, erythropoietin-stimulating agent.

Survival probabilities of the high-risk groups classified on the basis of RF_time_all and RF_time_v8 were lower than those of the low-risk groups (Table 2). The HRs of the primary outcome of the high-risk groups classified on the basis of RF_time_all and RF_time_v8 were 104.9 (95% CI 70.81, 155.3) and 90.9 (95% CI 62.29, 132.7), respectively. Likewise, the HRs of ESKD were 103.3 (95% CI 70.0, 152.4) and 89.6 (95% CI 61.6, 130.3), and the HRs of death were 14.85 (95% CI 9.75, 22.62) and 14.36 (95% CI 9.43, 21.86) for RF_time_all and RF_time_v8, respectively.

**Table 2:** Outcome events of risk groups classified on the basis of machine-learning models.

Model names	RF_time_all			RF_time_v8		
Risk groups	Low-risk group	High-risk group	*p* value	Low-risk group	High-risk group	*p* value
Primary outcome (%)	235 (0.9)	43 (2.9)	<0.0001	238 (0.9)	40 (2.7)	<0.0001
ESKD (%)	153 (0.6)	34 (2.3)	<0.0001	156 (0.6)	31 (2.1)	<0.0001
Death (%)	82 (0.3)	9 (0.6)	0.099	82 (0.3)	9 (0.6)	0.099
Follow-up period (days)	901 [527, 1,052]	710 [407, 1,038]	<0.0001	898 [526, 1052]	738.5 [428, 1043]	<0.0001

Continuous variables are shown as median (interquartile range). Categorical variables are shown as n (%). Abbreviations: ESKD, end-stage kidney disease.

### Implementation of the models in clinical settings

**Fig 4:**
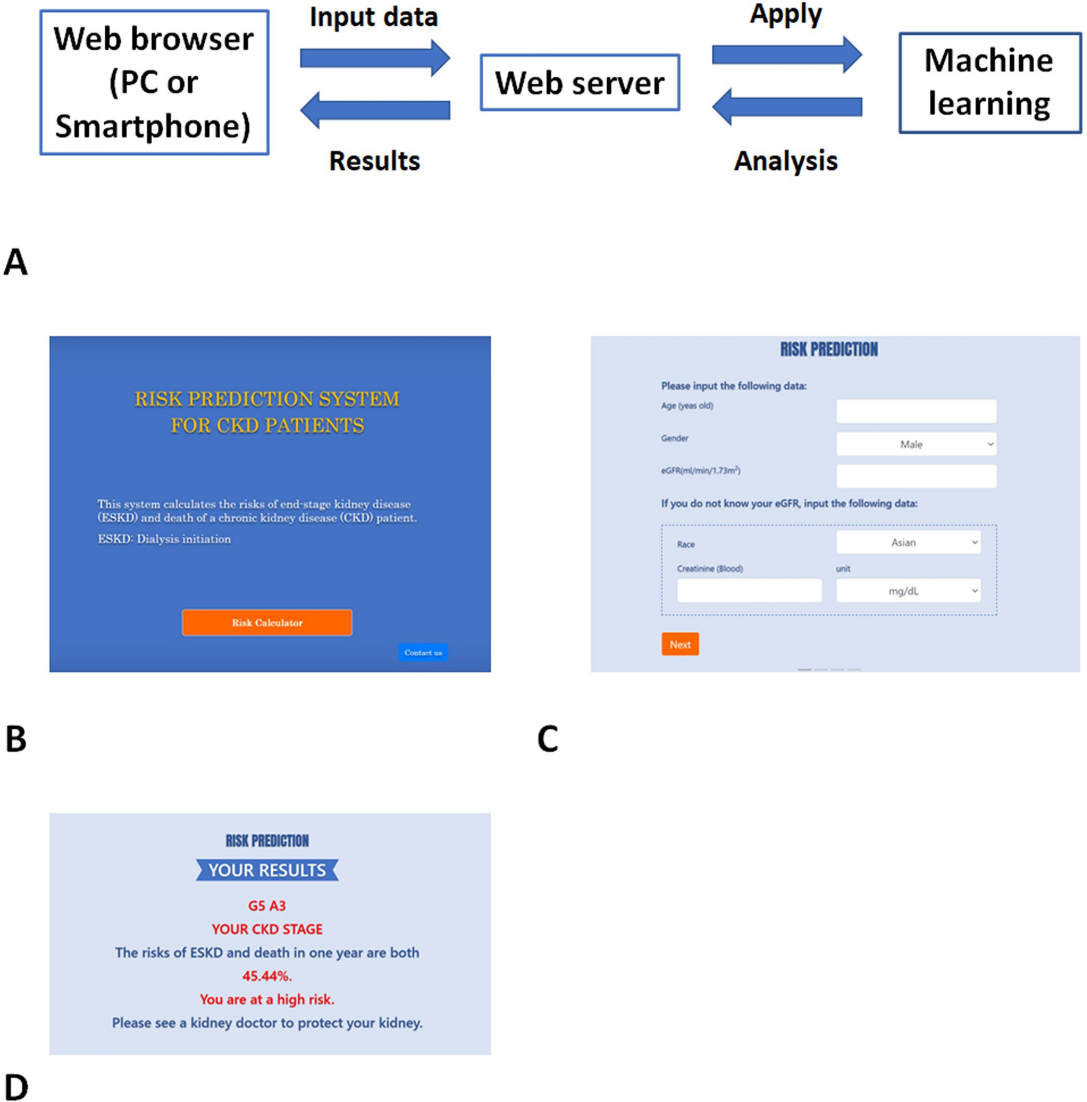
Images of Web pages of risk-prediction system. A. Conceptual design of the system: Patients’ data are input to the machine-learning models. The results are shown on the browser. B. Start page: Go to the Web site, http://160.16.88.112:8000/. C. Input page: Input data of a patient. D. Result page: The expected probability is shown with messages to focus the user’s attention on CKD. Abbreviation: CKD, chronic kidney disease.

We developed a Web-based risk-prediction system using RF_time_all and RF_time_v8. Then, we uploaded the system on the Web site for users to access and easily estimate CKD patients’ risk (http://160.16.88.112:8000/) (Fig 4).

## Discussion

In this study, we developed a Web-based risk-prediction system consisting of machine-learning models for the prediction of CKD patients’ renal and life prognoses. The machine-learning-based probability could accurately determine CKD patients’ prognoses, and the system was applicable with high accuracy to CKD patients with various conditions and comorbidities. Our findings indicated that the machine-learning-based probability can be a new risk index for CKD patients. Moreover, we developed a Web-based risk-prediction system using the two selected RF models to apply the predicted probability as an index in clinical practice. This system enabled easy and quick estimation of the prognoses of CKD patients, and it provided a recommendation that patients be referred to a specialist. To the best of our knowledge, this study is the first on the risk prediction of CKD patients using artificial intelligence (AI), whose performances were thoroughly tested and implemented. This would be a supportive tool for providing medical treatment to patients at high risks of outcome events.

Probabilities of the patients’ prognoses are useful as a medical index in clinical practice. Various risk-prediction models have been developed using statistical models to predict CKD progression [13–15]. KFRE was developed for CKD patients using Cox proportional hazards models, and the survival index was developed using logistic regression (LR) models by Dialysis Outcomes and Practice Patterns Study (DOPPS) for hemodialysis patients [16,22]. These probabilities can extract patients’ features as a single variable, such as a clinical propensity score, and make it easy to estimate the condition of patients by simply substituting values in the models.

Treatment plans are often selected while considering numerous trade-offs. For example, should a low-protein diet be prescribed to prevent hyperphosphatemia and CKD-MBD, or should it not be prescribed to prevent malnutrition/sarcopenia/frailty? In this situation, probabilities of patients’ prognoses will give us the candidate strategies and assist in the selection of suitable therapies [16]. Probabilities estimated using machine-learning models, which allow a much larger number of variables than statistical models, can be applied to a highly complicated situation involving follow-up conditions, drug selection, the determination of blood access for dialysis, and the initiation of dialysis and kidney transplantation. Probability-based machine-learning models have a wide range of applications in medicine.

It is often very difficult for medical staff to calculate statistical formulae including log or exponential functions with many indices during busy medical examinations. To enable busy medical staff and patients to access the system quickly and easily, our risk-prediction system was uploaded on a Web site. The system developed in this study was based on RF models in a server, which analyzed patient data at a high speed and could be easily accessed through the internet using PCs and smartphones. To decrease the burden of measurement and inputting patients’ data, the variables used in the models were universally assessed in clinical settings, and the number of variables in the models of our system was limited. AI and communication technologies were used to realize this system.

Our models are applicable to clinical settings within their limitations. First, the data were obtained in Japan. The accuracies of the models for Asian patients will be high, but the results obtained in non-Asian countries may be biased by the patient population. Transfer learning will improve the accuracy of this system for global use as described above. Second, the data did not sufficiently include those for assessing variables that were not included in EMRs, such as lifestyle, food intake, family history, and social determinants of health. Third, new drugs that can effectively prevent CKD progression, such as sodium-glucose cotransporter-2 inhibitors, have been developed [23,24]. In this study, we did not use data on those drugs, because it was only a year since it was approved for use in CKD therapy in Japan. It is necessary to wait until sufficient data on those drugs will be accumulated. Fourth, RF-related models were developed in this study. A deep learning model was not used because the number of variables was small and because deep learning models tend to overfit. Thus, RF-related models were suitable for this study. Fifth, EMR data were used in this study. Although such data reflect real-world settings, all of the clinical variables are not always measured. Variables rarely measured have many missing values and are difficult to use in machine-learning models. Moreover, there is a problem of when to measure variables with day-to-day variations, such as blood glucose level. Therefore, commonly used and stable variables which were used in this study. It is necessary to examine whether variables are appropriate for model development when real-world data are used. Sixth, the WEB system was developed in this observational study. An interventional study should be conducted to show the effects of this system on CKD therapy and improvement of CKD progression.

In conclusion, this study showed that the machine-learning-based Web system accurately predicts a CKD patient’s increased risk of ESKD or death and is useful as a new tool for clinical practice.

## Materials and methods

This study consisted of 4 steps; (1) model development, (2) model selection, (3) model validation, and (4) development of the WEB-based risk-prediction system. Steps (1) and (2) were conducted using data from EMRs of CKD patients. Step (3) was conducted using data from a cohort study of CKD patients.

### Study design and ethics

In this study, the machine-learning models were developed using data from EMRs of Kawasaki Medical School Hospital, Okayama, Japan, from January 1st, 2014 to December 31st, 2017 and validated using data from a cohort study of CKD patients who visited Kawasaki Medical School Hospital from January 1st, 2018 to December 31st, 2020 (CKD cohort study). This study was approved by the ethics committee of Kawasaki Medical School. Owing to the nature of this study, it was exempt from the need to obtain informed consent from participants (Nos. 3444, 3445, and 5306). The study was performed in accordance with the relevant guidelines and the Declaration of Helsinki of 1975 as revised in 1983.

### Step (1) Development of machine-learning models

#### Study population

The EMR data as a dataset were extracted from the server in Kawasaki Medical School Hospital. The dataset was preprocessed for analysis because it was composed of all data of all patients in a column. The dataset was divided by variables, and the separated data were merged for each patient and medical examination day in a table for analysis. The dataset contained time-series data of patients (time-series dataset n = 83,703) (S3 Fig).

The exclusion criteria were as follows: patients younger than 20 years, patients on any type of dialysis, and patients who had malignancies. The patients with malignancies often show a shorter survival than others and tend to die before they reach ESKD. Their disease courses are different from the typical course of CKD. If their data, which are not related to CKD, had been included among the data for machine learning, they may affect the performance of machine-learning models and variable selection. Therefore, patients with malignancies were excluded in this study. Moreover, patients who had missing baseline data were excluded from the dataset. Thus, the time-series data obtained on every medical examination day from 3,714 patients were used in the development of machine-learning models (time-series dataset n = 66,981). The median (IQR) number of repeatedly measured times per patient was 16 (7, 29). The first-visit data of a patient were treated as baseline data (baseline dataset n = 3,714).

In this study, two types of machine-learning model were developed using baseline data or time-series data. To develop and select the models, the data were further assigned into the development dataset (including 80% of the patients, baseline dataset n = 2,967, time-series dataset n = 31,763) and a selection dataset (baseline dataset n = 747) on the basis of patient ID number.

The development dataset contained baseline data and time-series data, each of which was further divided into two datasets on the basis of patient ID number. About 70% of the development dataset was used to train the models (baseline dataset n = 2,076, time-series dataset n = 22,234), and the rest was used to test the models (baseline dataset n = 891, time-series dataset n = 9,529). As a result, four datasets were prepared: the baseline dataset to train the models (baseline dataset n = 2,076), the time-series dataset to train the models (time-series dataset n = 22,234), the baseline dataset to test the models (baseline dataset n = 891), and the time-series dataset to test the models (time-series dataset n = 9,529). The two training datasets included the same patients as well as the two testing datasets.

On the other hand, the selection dataset contained only baseline data (baseline dataset n = 747). Baseline characteristics and outcome events are shown in S5 Table and S6 Table, respectively.

#### Variables

The variables were CKD-related factors and medications in the EMR data [4,25–28]. They were selected for global use on the basis of their being common measurements used at outpatient clinics and ease of data input by users at implementation. The variables with more than 20% of their time-series data missing were not included in the analysis, because these variables were considered not to be usually measured at clinical settings.

The following variables were used in the analysis as follows (S7 Table): age; gender; DM; hypertension; history of CVD; eGFR; serum albumin, sodium, potassium, calcium, phosphorus, low-density lipoprotein, and uric acid levels; white blood cell count; hemoglobin level; urinary protein-to-creatinine ratio (UPCR); and use of renin-angiotensin-aldosterone system inhibitors, phosphorus absorbents, vitamin D, statins, uric-acid-lowering medicines, and erythropoietin-stimulating agents. The primary outcome was ESKD or death. If no outcomes were observed within the follow-up period, the observation data were treated as censored data. The onset of ESKD was defined as the initiation of renal replacement therapy. None of the patients in this study had received a kidney transplantation.

eGFR was calculated using the equation for the Japanese population on the basis of the serum creatinine level measured using the enzymatic method [29]. The baseline data were defined as the first measurements within 30 days of the initial eGFR. KFRE was calculated using eGFR, age, gender, and natural logarithm (urine albumin-to-creatinine ratio, UACR) [14]. Negative values of KFRE were treated as zero. Because the Japanese national health insurance covers UACR measurement only for DM patients, UACR was estimated from the UPCR using Weaver *et al*.*’*s estimation formulae [30]. The last observation data carried forward were input in place of all missing time-series data.

#### Machine-learning models

The *scikit-learn* and *numpy* packages of Python were mainly used in model development. RF, Gradient Boosting Decision Tree (GB), and eXtreme Gradient Boosting (XG) models were developed using *RandomForestClassifier*, *GradientBoostingClassifier*, and *XGBClassifier* in *scikit-learn*, respectively. Their parameters were determined using a grid search algorithm, *GridSearchCV*, to search for the hyperparameters for the models, which were the number of estimators (trees), the maximum percentage of features used in each tree, maximum depth, and class weight. The parameters of *GridSearchCV* were the maximum number of trees of 1000, depth of 10, leaf of 10, and sample split of 10. The parameters of the selected models were as follows. RF_time_all: number of trees = 1000, maximum depth = 10, minimum samples leaf = 5, minimum samples split = 5; RF_time_v8: number of trees = 1000, max depth = 10, minimum samples leaf = 5, minimum samples split = 5. In the development of the models with selected variables, the variables were ranked by their permutation importance in each model using *numpy* and then selected. *pickle* was used to save and load these trained models.

#### Model development

We developed risk-prediction models to classify the primary outcome over 1 year using the RF, GB, and XG models, which were trained to predict the primary outcome. Each type of machine-learning model included all 22 variables or selected variables. Variables were selected by their permutation importance in each model (S4 Fig). The numbers of variables selected were 7 for RF_base, 8 for RF_time, 13 and 6 for GB_base, 7 for GB_time, 17 and 7 for XG_base, and 10 and 6 for XG_time.

A multivariate LR model was developed using the hierarchical backward elimination procedure. The LR model with 11 selected variables was developed (S8 Table).

#### Statistical analyses

Baseline characteristics and outcomes were compared between the development and selection datasets using the chi-square test, t-test, and Mann-Whitney U test, as appropriate. After the proportional hazards assumption was confirmed using a double logarithmic plot, multivariate Cox proportional hazards models were used to compare the risks of outcomes between groups. The results are presented here as HRs with 95% CIs. In the analysis of the competing risks of ESKD and death, we used Fine and Gray competing risk regression models. Statistical significance was defined as a two-sided *p*<0.05. All statistical analyses were carried out using SAS version 9.4 (SAS Institute, Cary, NC, USA), Python 3.8.2 (Python Software Foundation, DE, USA), and R version 3.6.1 (R Foundation for Statistical Computing, Vienna, Austria).

## Step (2) Model selection

### Dataset and accuracies of the risk prediction

After the models were developed, they were applied to the model selection dataset including the baseline data of 747 patients. To evaluate models, a dataset composed of 1,000 sampled datasets was generated from the model selection dataset by bootstrapping 1,000 times so that the C-statistics with 95% CIs for the prediction of the outcomes were evaluated.

Moreover, subgroup analyses were conducted. The C-statistics were assessed in the following relevant subgroups: high (60 mL/min/1.73m^2^ or higher) and low (less than 60 mL/min/1.73m^2^) eGFRs, DM status, and young (less than 65 years) and old (65 years or older) ages.

#### Risks of outcomes

A well-developed model is one for which the predicted probabilities strongly correlate with the risks of the outcomes. To evaluate the relationship between the probability predicted using the models and the risk of the primary outcome, univariate Cox proportional hazards models including the spline terms of the predicted probability were developed using the model selection dataset.

Moreover, for users to easily understand the severity of risks in a patient, cutoff levels of the predicted probabilities were determined for the categorization of patients. A cutoff level of each model was determined on the basis of the Youden index on the receiver operating characteristic curve of the prediction of the primary outcome over 1 year using the development dataset [31]. Then, the patients were categorized into high- and low-risk groups.

### Step (3) Validation of the machine-learning models using data from a cohort study

#### Study population

The machine-learning models were validated using a dataset of a cohort study different from the development dataset. The CKD cohort study was conducted for three years. CKD patients who regularly visited the outpatient clinic of Kawasaki Medical School were registered (n = 67,957) (S5 Fig). The exclusion criteria were the same as those for the dataset used for the model development. Moreover, the patients who had no eGFR data were also excluded. A total of 26,906 patients were included in the analysis and followed up during the study period. The data were extracted from the cohort dataset derived from an EMR server in Kawasaki Medical School Hospital. The data at the first visit of patients during the study period were treated as baseline data. The dataset contained baseline data only.

#### Variables

The names and definitions of the variables were the same as those for the dataset used for the model development (S7 Table). Multiple imputation was conducted to account for missing data in analyses. The primary outcome was defined as ESKD or death for the follow-up period. The data of patients who did not have either of these outcomes were treated as censored data. The probability of the primary outcome was estimated using the machine-learning models and used as a risk index.

#### Statistical analysis

The C-statistics and 95% CIs for the accuracies of the prediction of the outcomes, such as the primary outcome, ESKD, or death, of the estimated probability were evaluated using the 1000-times bootstrap method. The C-statistics of the models for the outcomes over 1 year were statistically compared with that of KFRE using the bootstrap method.

For the probabilities to be used in clinical settings, not only incident risk but also survival analysis considering a long follow-up period was required. Thus, univariate Cox proportional hazards models including the spline terms of the probability were used to evaluate the relationship between the probability and risk of the primary outcome.

Then, the patients were divided into two groups on the basis of the cutoff levels determined at Step (2). The hazard of the primary outcome in the high-risk group was compared with that in the low-risk group. In the analysis of the competing risks of ESKD and death, Fine and Gray competing risk regression models were examined.

### Step (4) Development of a Web-based risk-prediction system and its specifications

To deploy the machine-learning models on the Web for users to try, we developed a Web-based risk-prediction system. We set up a Web server and developed a Web system in which the models were applied at the backend of the system. The system was used as follows. (1) A user accesses the system through a Web browser on a PC or smartphone. (2) Entered user data are applied to machine-learning models through the server, which estimates the probability of the primary outcome. (3) The results and messages to the user are shown on the Web browser.

In this system, there are several formulae with which users can easily calculate the probability of the primary outcome. eGFR was calculated using the Chronic Kidney Disease Epidemiology Collaboration (CKD-EPI) equation for white and black people, and using the equation for the Japanese population for Asian people on the basis of the serum creatinine level measured using the enzymatic method [29,32]. This system minimizes prediction errors due to differences in the methods of measuring creatinine levels, because the risk probability was estimated on the basis of eGFR. Moreover, units can be selected for patient data, because the units of variables used in different countries vary.

## Supporting information

S1 Fig C-statistics of models for prediction of outcomes over 2 and 3 years at model selection stage. (PDF)

S2 Fig C-statistics of models for prediction of primary outcome over 1 year in subclasses at model selection stage. (PDF)

S3 Fig Study population for model development and selection. (PDF)

S4 Fig Ranks of variables in models. (PDF)

S5 Fig Study population for model validation. (PDF)

S1 Table Summary of C-statistics of models at model selection stage. (PDF)

S2 Table Baseline characteristics of patients used as dataset for model validation. (PDF)

S3 Table Outcome events of model validation dataset. (PDF)

S4 Table Summary of model performances at model validation stage. (PDF)

S5 Table Baseline characteristics of model development and selection datasets. (PDF)

S6 Table Outcome events of model development and selection datasets. (PDF)

S7 Table Definition of variables. (PDF)

S8 Table Selected variables in logistic regression model and effect of each variable on primary outcome. (PDF)
