# Supplementary material for: Machine-learning-based Web system for the prediction of chronic kidney disease progression and mortality
Source: PLOS Digit Health. 2023 Jan 18;2(1):e0000188. doi: 10.1371/journal.pdig.0000188 (PMC9931312; doi:10.1371/journal.pdig.0000188)
Supplement: S1 Fig — (PDF) [file pdig.0000188.s001.pdf]

S1 Fig. C-statistics of models for prediction of outcomes over 2 and 3 years at model selection stage.

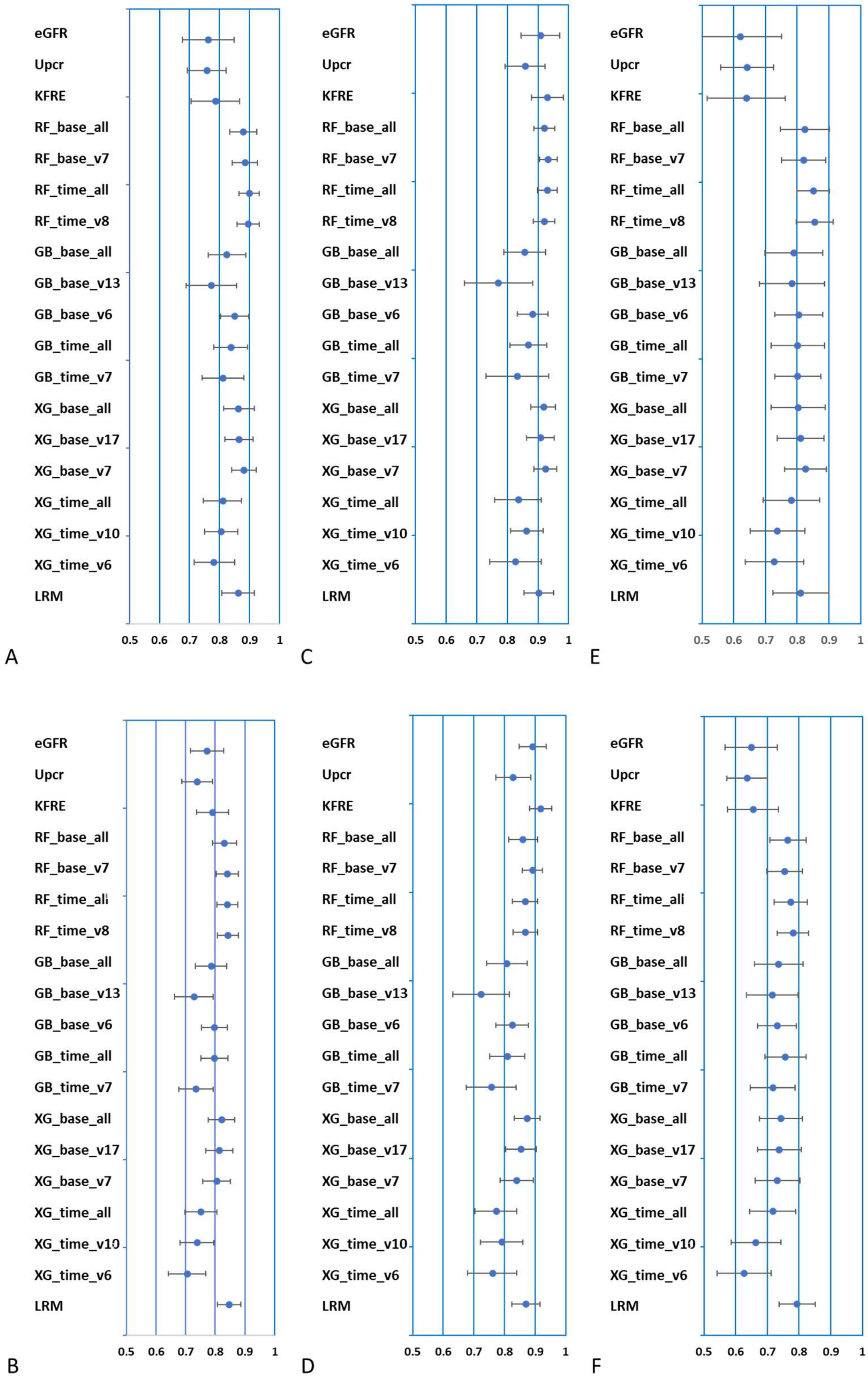

Values show C-statistics with 95% CIs.

A: C-statistics for prediction of the primary outcome over 2 years.

B: C-statistics for prediction of the primary outcome over 3 years.

C: C-statistics for prediction of ESKD over 2 years.

D: C-statistics for prediction of ESKD over 3 years.

E: C-statistics for prediction of death over 2 years.

F: C-statistics for prediction of death over 3 years.

Abbreviation: eGFR, estimated glomerular filtration rate; UPCR, urinary protein-to-creatinine ratio; KFRE, kidney failure risk equation; RF, Random Forest; GB, Gradient Boosting Decision Tree; XG, eXtreme Gradient Boosting; LRM, logistic regression model.
