# Supplementary material for: Machine-learning-based Web system for the prediction of chronic kidney disease progression and mortality
Source: PLOS Digit Health. 2023 Jan 18;2(1):e0000188. doi: 10.1371/journal.pdig.0000188 (PMC9931312; doi:10.1371/journal.pdig.0000188)
Supplement: S2 Fig — (PDF) [file pdig.0000188.s002.pdf]

S2 Fig. C-statistics of models for prediction of primary outcome over 1 year in subclasses at model selection stage.

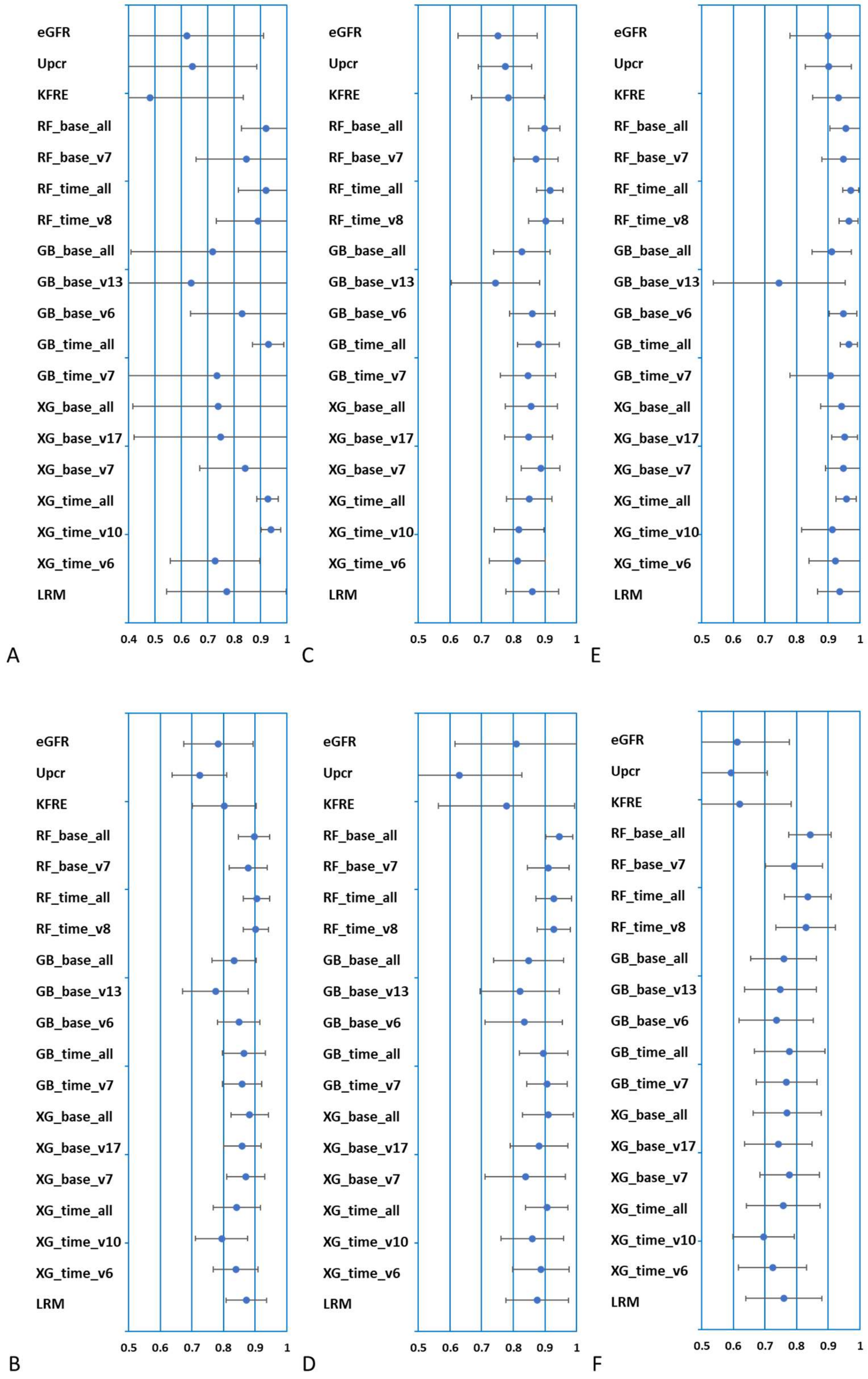

Values show C-statistics with 95% CIs.

A: High eGFR (eGFR 60 mL/min/1.73m^2 or higher).

B: Low eGFR (eGFR less than 60 mL/min/1.73m^2).

C: Non-DM.

D: DM.

E: Young (younger than 65 years).

F: Old (65 years or older).

Abbreviation: eGFR, estimated glomerular filtration rate; UPCR, urinary protein-to-creatinine ratio; KFRE, kidney failure risk equation; RF, Random Forest; GB, Gradient Boosting Decision Tree; XG, eXtreme Gradient Boosting; LRM, logistic regression model; DM, diabetes mellitus.
