# Supplementary material for: Machine-learning-based Web system for the prediction of chronic kidney disease progression and mortality
Source: PLOS Digit Health. 2023 Jan 18;2(1):e0000188. doi: 10.1371/journal.pdig.0000188 (PMC9931312; doi:10.1371/journal.pdig.0000188)
Supplement: S3 Fig — (PDF) [file pdig.0000188.s003.pdf]

S3 Fig. Study population for model development and selection.

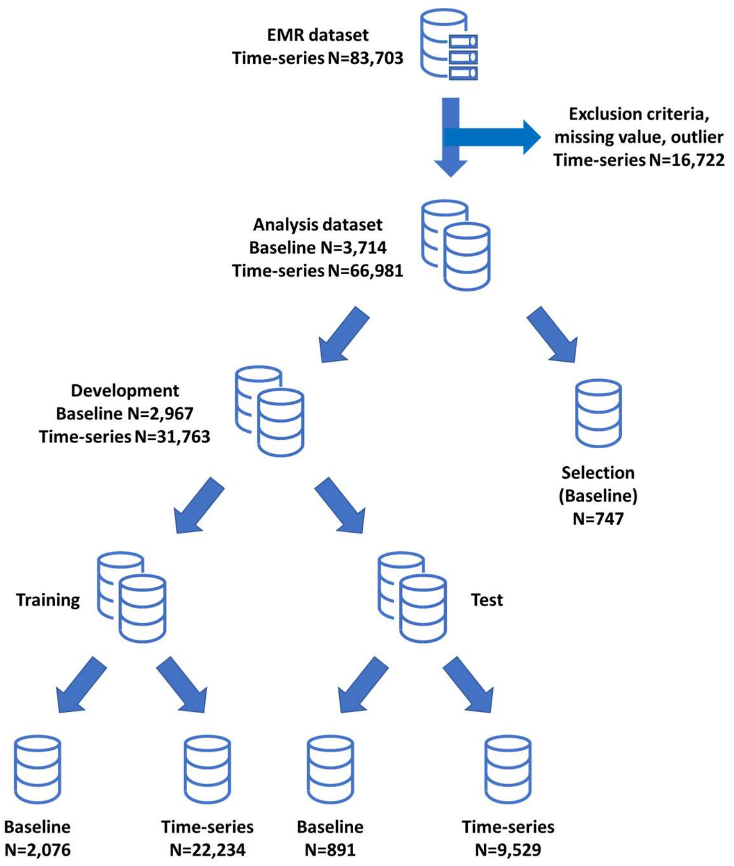


EMR data included baseline and time-series data. The analysis data were randomly divided into the development and selection datasets on the basis of patient ID number. The development dataset was further divided randomly into the training and test datasets on the basis of patient ID number. Thus, the training datasets of baseline and time-series dataset contained the same patients. Similarly, the patients in the test baseline dataset were the same as these in the time-series dataset. The dataset used for model selection was composed of baseline data.

Abbreviations: Baseline, baseline data; Time-series, time-series data on every medical examination day.
