# Supplementary material for: Machine-learning-based Web system for the prediction of chronic kidney disease progression and mortality
Source: PLOS Digit Health. 2023 Jan 18;2(1):e0000188. doi: 10.1371/journal.pdig.0000188 (PMC9931312; doi:10.1371/journal.pdig.0000188)
Supplement: S4 Fig — (PDF) [file pdig.0000188.s004.pdf]

S4 Fig. Ranks of variables in models.

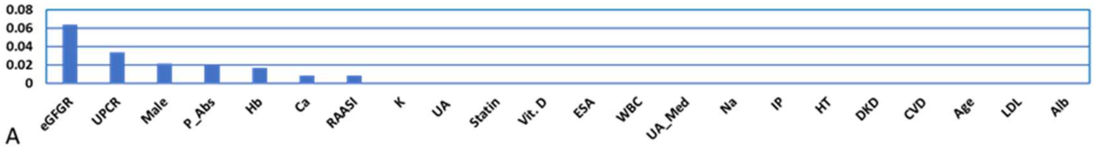

A

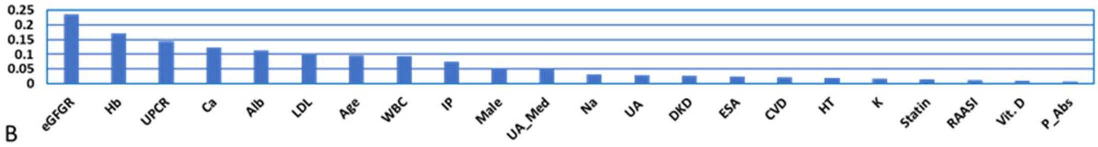

B

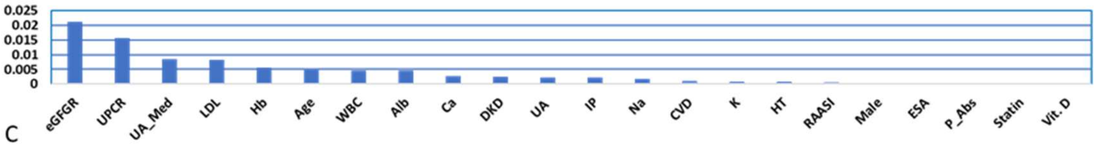

C

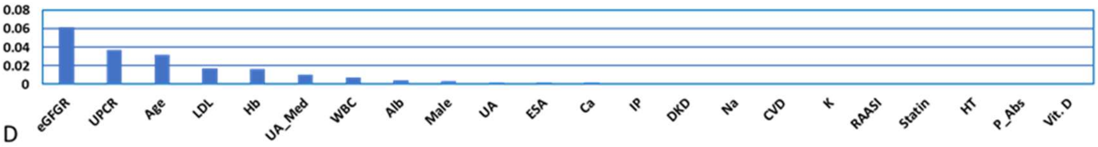

D

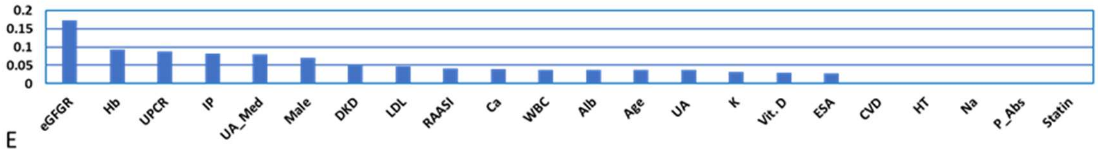

E

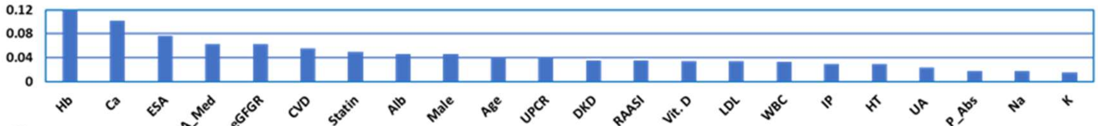

F

The values of the variables show their permutation importance in the models. The names of the variables are shown in order of their importance.

A RF_base_all

B RF_time_all

C GB_base_all

D GB_time_all

E XG_base_all

F XG_time_all

Variable names are shown in S6 table.

Abbreviations: RF, random forest; GB, Gradient Boosting Decision Tree; XG, eXtreme Gradient Boosting.
