# Supplementary material for: Machine-learning-based Web system for the prediction of chronic kidney disease progression and mortality
Source: PLOS Digit Health. 2023 Jan 18;2(1):e0000188. doi: 10.1371/journal.pdig.0000188 (PMC9931312; doi:10.1371/journal.pdig.0000188)
Supplement: S5 Fig — (PDF) [file pdig.0000188.s005.pdf]

S5 Fig. Study population for model validation.

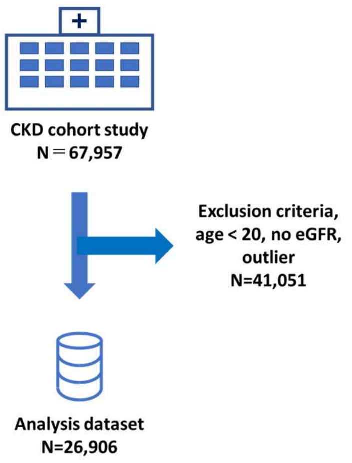

Data from the CKD cohort study were used to validate the machine-learning models. This dataset contained baseline data of patients.
