# Supplementary material for: Machine-learning-based Web system for the prediction of chronic kidney disease progression and mortality
Source: PLOS Digit Health. 2023 Jan 18;2(1):e0000188. doi: 10.1371/journal.pdig.0000188 (PMC9931312; doi:10.1371/journal.pdig.0000188)
Supplement: S1 Table — (PDF) [file pdig.0000188.s006.pdf]

S1 Table. Summary of C-statistics of models at model selection stage.

Model name	Primary outcome, C>0.8	ESKD, C>0.8	Death, C>0.7	eGFR≥60, C>0.75	eGFR<60, C>0.8	Non-DM, C>0.9	DM, C>0.8	Young, C>0.9	Old, C>0.8	Number of Yes entries
RF_base_all	Yes	Yes	Yes	Yes	Yes	Yes	Yes		Yes	8
RF_base_v7	Yes	Yes	Yes		Yes	Yes	Yes		Yes	7
RF_time_all	Yes	Yes	Yes	Yes	Yes	Yes	Yes	Yes	Yes	9
RF_time_v8	Yes	Yes	Yes		Yes	Yes	Yes	Yes	Yes	8
GB_base_all		Yes	Yes							2
GB_base_v13		Yes								1
GB_base_v6								Yes		1
GB_time_all	Yes	Yes	Yes	Yes		Yes	Yes	Yes		7
GB_time_v7		Yes	Yes				Yes			3
XG_base_all	Yes	Yes			Yes		Yes			4
XG_base_v17		Yes			Yes			Yes		3
XG_base_v7	Yes	Yes	Yes		Yes	Yes				5
XG_time_all	Yes	Yes	Yes	Yes			Yes	Yes		6
XG_time_v10		Yes		Yes						2
XG_time_v6		Yes								1
LRM		Yes			Yes					2

If a model meets a requirement, "Yes" is filled in a cell. The total number of cells with "Yes" is counted in the right column. C-statistics of a model greater than 0.7 to 0.9 shows that they were statistically significantly high (p<0.05).

Abbreviations: C, C-statistics; ESKD, end-stage kidney disease; DM, diabetes mellitus; RF, Random Forest; GB, Gradient Boosting Decision Tree; XG, eXtreme Gradient Boosting; LRM, logistic regression model.
