# Supplementary material for: Machine-learning-based Web system for the prediction of chronic kidney disease progression and mortality
Source: PLOS Digit Health. 2023 Jan 18;2(1):e0000188. doi: 10.1371/journal.pdig.0000188 (PMC9931312; doi:10.1371/journal.pdig.0000188)
Supplement: S2 Table — (PDF) [file pdig.0000188.s007.pdf]

S2 Table. Baseline characteristics of patients used as dataset for model validation.

N	26,906
**Demographic characteristics**	
Age (years)	61.2±16.4
Male (%)	13,779 (51.2)
Comorbidities	
DM (%)	5,637 (21.0)
Hypertension (%)	5,570 (20.7)
CVD (%)	189 (0.7)
**Laboratory data**	
eGFR (mL/min/1.73m^2)	73.1 ± 30.9
Albumin (g/dL)	4.2±0.5
Sodium (mmol/L)	140.3±2.6
Potassium (mmol/L)	4.2±0.4
Calcium (mg/dL)	9.2±0.5
Phosphorus (mg/dL)	3.4±0.7
LDL (mg/dL)	111.0±32.3
Uric acid (mg/dL)	5.2±1.5
WBC (10^3/μL)	6.3±3.1
Hemoglobin (g/dL)	13.6±1.9
UPCR (g/gCre)	0.28 [0.11, 1.05]
**Medications**	
RAASI (%)	4,675 (17.4)
Phosphorus absorbent (%)	261 (1.0)
Vitamin D (%)	716 (2.7)
Statin (%)	4,342 (16.1)
Uric-acid-lowering medicines (%)	2,446 (9.1)
ESA (%)	747 (2.8)

In the cohort, the number of patients aged 65 years and older was 13,047 (48.5%). The distribution of patients in different CKD stages was as follows: G1, 5,044 (18.7%); G2, 15,134 (56.2%); G3a, 4,274 (15.9%); G3b, 1,445 (5.4%); G4, 536 (2.0%); and G5, 473 (1.8%).

Continuous variables are shown as mean±SD or median (interquartile range). Categorical

variables are shown as n (%).

Abbreviations: DM, diabetes mellitus; CVD, cardiovascular disease; eGFR, estimated glomerular filtration rate; LDL, low-density lipoprotein; WBC, white blood cells; UPCR, urinary protein-to-creatinine ratio; RAASI, renin-angiotensin-aldosterone system inhibitor; ESA, erythropoietin-stimulating agent.
