# Supplementary material for: Machine-learning-based Web system for the prediction of chronic kidney disease progression and mortality
Source: PLOS Digit Health. 2023 Jan 18;2(1):e0000188. doi: 10.1371/journal.pdig.0000188 (PMC9931312; doi:10.1371/journal.pdig.0000188)
Supplement: S3 Table — (PDF) [file pdig.0000188.s008.pdf]

S3 Table. Outcome events of model validation dataset.

N	26,906
Primary outcome (%)	278 (1.0)
ESKD (%)	187 (0.7)
Death (%)	91 (0.3)
Follow-up period (days)	894 [519, 1052]

Continuous variables are shown as median (interquartile range). Categorical variables are shown as n (%).

Abbreviation: ESKD, end-stage kidney disease.
