# Supplementary material for: Machine-learning-based Web system for the prediction of chronic kidney disease progression and mortality
Source: PLOS Digit Health. 2023 Jan 18;2(1):e0000188. doi: 10.1371/journal.pdig.0000188 (PMC9931312; doi:10.1371/journal.pdig.0000188)
Supplement: S4 Table — (PDF) [file pdig.0000188.s009.pdf]

S4 Table. Summary of model performances at model validation stage.

Model name	Primary outcome, C>0.8	ESKD, C>0.8	Death, C>0.7	eGFR≥60, C>0.75	eGFR<60, C>0.8	Non-DM, C>0.9	DM, C>0.8	Young, C>0.9	Old, C>0.8	Number of Yes entries
RF_time_all	Yes	Yes	Yes	Yes	Yes	Yes	Yes	Yes	Yes	9
RF_time_v8	Yes	Yes	Yes	Yes	Yes	Yes	Yes	Yes	Yes	9

If a model meets a requirement, “Yes” is filled in a cell. The total number of cells with “Yes” is counted in the right column. C-statistics of a model greater than 0.7 to 0.9 shows that they were statistically significantly high ($p<0.05$).

Abbreviation: C, C-statistics; ESKD, end-stage kidney disease; DM, diabetes mellitus; RF, Random Forest.
