# Supplementary material for: Machine-learning-based Web system for the prediction of chronic kidney disease progression and mortality
Source: PLOS Digit Health. 2023 Jan 18;2(1):e0000188. doi: 10.1371/journal.pdig.0000188 (PMC9931312; doi:10.1371/journal.pdig.0000188)
Supplement: S5 Table — (PDF) [file pdig.0000188.s010.pdf]

S5 Table. Baseline characteristics of model development and selection datasets.

	All	Development	Selection	*p* value
N	3,714	2,967	747	
**Demographic characteristics**				
Age (years)	60.1±17.6	60.1±17.7	60.0±17.4	0.88
Male (%)	1,971 (53.1)	1,575 (53.1)	396 (53.0)	0.99
Comorbidities				
DM (%)	858 (23.1)	685 (23.1)	173 (23.2)	0.96
Hypertension (%)	1,881 (50.6)	1,510 (50.9)	371 (49.7)	0.59
CVD (%)	363 (9.8)	281 (9.5)	82 (11.0)	0.22
**Laboratory data**				
eGFR (mL/min/1.73m^2)	54.2±30.7	54.5±31.1	52.8±30.0	0.19
Albumin (g/dL)	3.9±0.7	3.9±0.7	3.9±0.7	0.23
Sodium (mmol/L)	140.3±3.1	140.4±3.0	140.2±3.4	0.34
Potassium (mmol/L)	4.4±0.6	4.4±0.6	4.4±0.6	0.60
Calcium (mg/dL)	9.0±0.7	9.0±0.7	8.9±0.7	0.65
Phosphorus (mg/dL)	3.5±0.9	3.5±0.9	3.5±0.9	0.64
LDL (mg/dL)	110.1±36.1	110.0± 35.3	110.6±39.0	0.66
Uric acid (mg/dL)	5.9±1.7	5.9±1.7	5.9±1.7	0.52
WBC (10^3/μL)	6.9±4.7	6.6±3.3	6.9±3.1	0.27
Hemoglobin (g/dL)	12.9±2.3	13.0±2.3	12.9±2.2	0.62
UPCR (g/gCre)	1.69±3.02 0.50 [0.14, 1.79]	1.69±3.02 0.49 [0.14, 1.80]	1.71±3.03 0.53 [0.13, 1.74]	0.73
**Medications**				
RAASI (%)	983 (26.5)	802 (27.0)	181 (24.3)	0.14
Phosphorus absorbent (%)	101 (2.7)	79 (2.7)	22 (2.9)	0.76
Vitamin D (%)	419 (11.3)	334 (11.3)	85 (11.4)	0.90
Statin (%)	939 (25.3)	746 (25.1)	193 (25.8)	0.67
Uric-acid-lowering medicines (%)	758 (20.4)	618 (20.8)	140 (18.7)	0.22
ESA (%)	508 (13.7)	392 (13.2)	116 (15.5)	0.11

Continuous variables are shown as mean±SD or median (interquartile range). Categorical variables are shown as n (%).

The number of patients aged 65 years and older was 1,601 (43.1%). The distribution of patients in different CKD stages was as follows: G1, 430 (11.6%); G2, 804 (21.7%); G3a, 1,007 (27.11%); G3b, 660 (17.8%); G4, 468 (12.6%); and G5, 345 (9.3%). There were no statistically significant differences in the baseline characteristics between the development and selection datasets.

Abbreviations: Development, dataset for model development; Selection, dataset for model selection; DM, diabetes mellitus; CVD, cardiovascular disease; eGFR, estimated glomerular filtration rate; LDL, low-density lipoprotein; WBC, white blood cells; UPCR, urinary protein-to-creatinine ratio; RAASI, renin-angiotensin-aldosterone system inhibitor; ESA, erythropoietin-stimulating agent.
