# Supplementary material for: Machine-learning-based Web system for the prediction of chronic kidney disease progression and mortality
Source: PLOS Digit Health. 2023 Jan 18;2(1):e0000188. doi: 10.1371/journal.pdig.0000188 (PMC9931312; doi:10.1371/journal.pdig.0000188)
Supplement: S6 Table — (PDF) [file pdig.0000188.s011.pdf]

S6 Table. Outcome events of model development and selection datasets.

	All	Development	Selection	*p* value
Primary outcome over 1 year (%)	190 (5.1)	156 (5.3)	34 (4.6)	0.52
Primary outcome over 2 years (%)	285 (7.7)	238 (8.0)	47 (6.3)	0.12
Primary outcome over 3 years (%)	454 (12.2)	367 (12.4)	87 (11.6)	0.66
ESKD over 1 year (%)	103 (2.8)	82 (2.8)	21 (2.8)	0.90
ESKD over 2 years (%)	155 (4.2)	127 (4.3)	28 (3.7)	0.61
ESKD over 3 years (%)	222 (6.0)	174 (5.9)	48 (6.4)	0.55
Death over 1 year (%)	87 (2.3)	74 (2.5)	13 (1.7)	0.54
Death over 2 years (%)	130 (3.5)	111 (3.7)	19 (2.5)	0.18
Death over 3 years (%)	232 (6.2)	193 (6.5)	39 (5.2)	0.42
Follow-up period (days)	774.3±413.6<br>1095 (387, 1095)	776.6±413.0<br>1095 (392.0, 1095)	765.2±416.4<br>1095 (372.5, 1095)	0.47

Continuous variables are shown as (interquartile range). Categorical variables are shown as n (%).

Abbreviations: Development, dataset for model development; Selection, dataset for model selection; ESKD, end-stage kidney disease.
