# Supplementary material for: Machine-learning-based Web system for the prediction of chronic kidney disease progression and mortality
Source: PLOS Digit Health. 2023 Jan 18;2(1):e0000188. doi: 10.1371/journal.pdig.0000188 (PMC9931312; doi:10.1371/journal.pdig.0000188)
Supplement: S7 Table — (PDF) [file pdig.0000188.s012.pdf]

S7 Table. Definition of variables.

Variable	Definition	Unit
Age	Continuous variable. Age.	years
Male	Binomial variable. Male.	0, 1
DKD	Binomial variable. Diabetes mellitus.	0, 1
HT	Binomial variable. Hypertension.	0, 1
CVD	Binomial variable. Cardiovascular disease.	0, 1
eGFGR	Continuous variable. eGFR.	$mL/min/1.73m^2$
Alb	Continuous variable. Albumin.	g/dL
Na	Continuous variable. Sodium.	mmol/L
K	Continuous variable. Potassium.	mmol/L
Ca	Continuous variable. Calcium.	mg/dL
IP	Continuous variable. Phosphorus.	mg/dL
LDL	Continuous variable. LDL.	mg/dL
UA	Continuous variable. Uric acid.	mg/dL
WBC	Continuous variable. WBC.	$10^3/\mu L$
Hb	Continuous variable. Hemoglobin.	g/dL
UPCR	Continuous variable. UPCR.	g/gCre
RAASI	Binomial variable. Use of RAASI	0, 1
P_Abs	Binomial variable. Use of phosphorus absorbent.	0, 1
Vit_D	Binomial variable. Use of vitamin D	0, 1
Statin	Binomial variable. Use of statin.	0, 1
UA_Med	Binomial variable. Use of uric-acid-lowering medicines.	0, 1
ESA	Binomial variable. Use of ESA.	0, 1

The names and definitions of variables are the same as those in the datasets used for the model development and validation.

Abbreviations: DKD, diabetes kidney disease; HT, hypertension; CVD, cardiovascular disease; eGFR, estimated glomerular filtration rate; LDL, low-density lipoprotein; UA, uric acid; WBC, white blood cell; UPCR, urinary protein-to-creatinine ratio; RAASI, renin angiotensin aldosterone system inhibitor; ESA, erythropoietin-stimulating agent.
