# Supplementary material for: Machine-learning-based Web system for the prediction of chronic kidney disease progression and mortality
Source: PLOS Digit Health. 2023 Jan 18;2(1):e0000188. doi: 10.1371/journal.pdig.0000188 (PMC9931312; doi:10.1371/journal.pdig.0000188)
Supplement: S8 Table — (PDF) [file pdig.0000188.s013.pdf]

S8 Table. Selected variables in logistic regression model and effect of each variable on primary outcome.

	Odds ratio	95% CI	*p* value
Age (1 year increase)	1.02	(1.01, 1.04)	0.0003
Male (ref. Female)	2.48	(1.68, 3.66)	<0.0001
DM (ref. non-DM)	1.54	(1.05, 2.27)	0.026
eGFR (1 mL/min/1.73m^2 increase)	0.991	(0.982, 0.999)	0.036
Potassium (1 mmol/L increase)	0.624	(0.473, 0.823)	0.0009
Calcium (1 mg/dL increase)	0.73	(0.559, 0.952)	0.020
Phosphorus (1 mg/dL increase)	1.53	(1.29, 1.82)	<0.0001
WBC (10^3/µL increase)	1.11	(1.05, 1.17)	<0.0001
Hemoglobin (1 g/dL increase)	0.713	(0.648, 0.784)	<0.0001
UPCR (1 g/gCre increase)	1.07	(1.03, 1.12)	<0.0012
Usage of Vitamin D, yes (ref. no)	2.12	(1.37, 3.29)	0.0008

The variables were selected on the basis of medical evidence and statistical significance.
Abbreviation: CI, confidence interval; DM, diabetes mellitus; eGFR, estimated glomerular filtration rate; WBC, white blood cells; UPCR, urinary protein-to-creatinine ratio.
